# Emerging Multiscale Biofabrication Approaches for Bacteriotherapy

**DOI:** 10.3390/molecules29020533

**Published:** 2024-01-22

**Authors:** Roberta Rovelli, Beatrice Cecchini, Lorenzo Zavagna, Bahareh Azimi, Claudio Ricci, Semih Esin, Mario Milazzo, Giovanna Batoni, Serena Danti

**Affiliations:** 1 Department of Civil and Industrial Engineering, University of Pisa, 56122 Pisa, Italybahareh.azimi@ing.unipi.it (B.A.);; 2 PEGASO Doctoral School of Life Sciences, University of Siena, 53100 Siena, Italy; l.zavagna@student.unisi.it; 3 Department of Translational Research and New Technologies in Medicine and Surgery, University of Pisa, 56126 Pisa, Italy; semih.esin@unipi.it (S.E.); giovanna.batoni@unipi.it (G.B.)

**Keywords:** 3D printing, electrospinning, electrospray, probiotics, tissue engineering, polysaccharides, sodium alginate

## Abstract

Bacteriotherapy is emerging as a strategic and effective approach to treat infections by providing putatively harmless bacteria (i.e., probiotics) as antagonists to pathogens. Proper delivery of probiotics or their metabolites (i.e., post-biotics) can facilitate their availing of biomaterial encapsulation via innovative manufacturing technologies. This review paper aims to provide the most recent biomaterial-assisted strategies proposed to treat infections or dysbiosis using bacteriotherapy. We revised the encapsulation processes across multiscale biomaterial approaches, which could be ideal for targeting different tissues and suit diverse therapeutic opportunities. Hydrogels, and specifically polysaccharides, are the focus of this review, as they have been reported to better sustain the vitality of the live cells incorporated. Specifically, the approaches used for fabricating hydrogel-based devices with increasing dimensionality (D)—namely, 0D (i.e., particles), 1D (i.e., fibers), 2D (i.e., fiber meshes), and 3D (i.e., scaffolds)—endowed with probiotics, were detailed by describing their advantages and challenges, along with a future overlook in the field. Electrospinning, electrospray, and 3D bioprinting were investigated as new biofabrication methods for probiotic encapsulation within multidimensional matrices. Finally, examples of biomaterial-based systems for cell and possibly post-biotic release were reported.

## 1. Introduction

**Figure 1:**
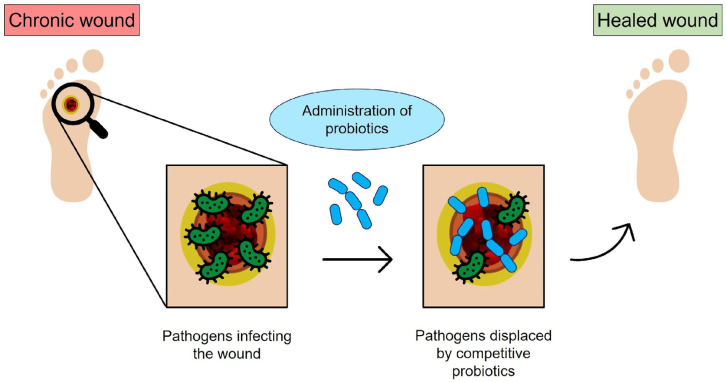
Schematic of bacteriotherapy applied to chronic wounds: the open wound is subjected to bacterial infection; however, the probiotics administered to the infected site can compete with pathogens and promote healing (original image by the authors).

The identification and implementation of alternative antimicrobial strategies that can replace or complement the use of antibiotics represent a widely recognized priority in the era of antimicrobial resistance. Hence, the identification of alternative therapeutic strategies that can replace or support the use of antibiotics has widely emerged as a priority in research [1]. Among different approaches, bacteriotherapy has emerged as an intriguing option. Bacteriotherapy relies on the use of harmless bacteria (e.g., probiotics) to compete with pathogens to displace them, suppress the expression of their virulence factors, and contrast the microbial ability to induce tissue injury and chronic inflammation [1,2,3]. The interest in bacteriotherapy to prevent or cure infectious diseases has recently been raised due to the rapid progression in molecular technologies. In particular, metagenomics sequencing tools have revealed the existence of body site-specific beneficial microbiota, whose dysbiosis is often associated with several infectious and non-infectious human disorders [4]. Thus, the reconstitution of site-specific microbiota via the administration of beneficial microbes might have a curative effect. A schematization of the working principle of bacteriotherapy is depicted in Figure 1.

According to the World Health Organization/Food and Agricultural Organization (WHO/FAO), probiotics are defined as living microorganisms that confer a health benefit to the host when administered in an adequate amount [5]. *Lactobacilli* and *Bifidobacteria* have been the best clinically documented and most commonly commercialized probiotic genera [2]. However, the list of species with potential health benefits is rapidly expanding, and novel probiotics are still under evaluation for future uses. Although the employment of bacteriotherapy has mainly concerned the treatment of gastrointestinal (GI) disorders and the reconstitution of a health-associated flora following dysbiosis of the urogenital tract, other types of applications are currently under investigation, including the use of such an approach for curing difficult-to-treat chronic wounds and respiratory airway infections [2,6,7].

For instance, the formation of diabetic foot ulcers (DFUs), a common type of chronic wound, has been recently reported to be favored by a substantial dysbiosis of the natural skin microbiota, which not only induces the emergence of the infection but also supports its evolution to chronicity. About 15–20% of ~420 million diabetic patients worldwide develop skin wounds across their lifespan, and the majority evolve into chronicity. This is a consequence of the diabetic-related impairment that thwarts an adequate immune response against a high microbial burden (both foreign microbes and commensal skin microbiota) [4]. DFUs are very difficult to treat, and hospitalization is often required to implement direct therapeutic procedures and patient care. To date, the most frequently applied strategies to heal diabetic foot ulcers have included the debridement of compromised tissues, the use of specialized dressings, and the administration of wide-spectrum antibiotics. However, success rates are still far from being satisfactory, and hospitalization and amputation are often required [4]. Therefore, new treatment approaches for this life-threatening problem must be urgently identified, and bacteriotherapy has been considered as an attractive option. Among the bacterial strains selected from the normal skin microbiota, *Staphylococcus epidermidis* is a promising candidate to treat skin diseases due to its capability to induce immune response, prevent microbe growth, and promote tissue repair [8,9].

Another interesting application of bacteriotherapy, which has been gaining increasing interest over the last few years, is the treatment of respiratory tract-related diseases. In this context, a powerful example is provided by the otitis media (OM), a pathology that has been associated with a long-term perturbation of the upper respiratory tract (URT)-specific microbiota. The OM is a URT infectious disease characterized by a perforated tympanic membrane with persistent drainage from the middle ear [10]. Accumulation of middle ear fluids gives the framework to create an ideal environment for bacterial translocation and growth and, ultimately, to the advent of inflammation [11]. The OM is a multifactorial condition most often encountered in children, whose first OM episode is usually experienced early, between 6 and 12 months. Among the different forms of OM, chronic otitis media (COM) is the most severe condition since it can last more than 3 months and develop recurrences across lifetimes. To date, OM is commonly treated with antibiotic prescriptions that, however, can cause harmful effects like diarrhea, vomiting, or skin rash [10]. Additionally, the impact of antibiotics on hearing is still unclear, and when most aggressive OM forms occur, there is no evidence that the administration of antibiotics can reduce the necessity of surgery, e.g., the placement of ventilation tubes into the tympanic membrane [11,12]. Therefore, new therapeutic strategies are required to prevent OM and reduce the need for surgical interventions in the case of COM. In view of this, the intake of harmless bacteria has been recently explored due to probiotics’ potential health benefits for URT diseases [10]. Lactic acid bacteria (LAB), already largely administered to treat GI issues, are among the most interesting probiotic candidates for the URT. Both oral and topical intake of LAB probiotics have been explored. The oral administration route aims at enhancing the immune responses systemically, mainly via GI immune cells, while the topical application of probiotics, e.g., nasal spray, allows to directly target the OM pathogens and exert a more effective antimicrobial activity. However, such an administration route has only been explored for a limited number of probiotic strains so far, and the acquired data are still unclear [13,14]. 

Probiotic administration is also proposed as a possible option for lower respiratory tract (LRT) infections. There are pathologic conditions in which patients are constantly prone to develop infections, like cystic fibrosis (CF), or are not eligible or not responsive to antibiotic treatments, which could greatly benefit from innovative antimicrobial strategies to substitute or complement antibiotic use [15]. Probiotic administration to CF patients via the oral route was able to partially restore gut dysbiosis, reduce intestinal inflammation, and lower lung through mechanisms that could be related by some studies to the gut–lung axis [1,16]. Administering probiotics through the respiratory, i.e., via nasal spray or aerosol, instead of through the GI route represents an expectant approach. It is believed that probiotic efficacy could be enhanced by their direct delivery at the infectious site, where they would compete with pathogens for adhering to lung mucosa, interfere with pathogen growth and virulence, improve the integrity of the lung mucosal barrier, and provide an immune-modulatory response [1]. Such a strategy was successful in mice and is under evaluation in humans [17,18,19,20].

Despite bacteriotherapy being considered a valuable option to prevent or treat various diseases, conflicting data on its real therapeutic efficacy are also present in the literature. Controversies concern not only the administered probiotic type and their dosage but also the best vehicles for bacteria delivery to the target site [10]. The delivery of bacterial cells to a target site takes great advantage of the use of biomaterials and devices, which preserve, confine, and release, at a controlled rate, the cells and/or their metabolites; thus, bacteriotherapy often embraces fabrication techniques.

This review paper aims to deliver a comprehensive description of the current multiscale fabrication approaches for bacteriotherapy, highlighting the advantages and disadvantages of each methodology, as well as providing a future overlook in this strategic and timely field.

## 2. Biofabrication Approaches

Biofabrication is emerging as a fascinating strategy to manufacture live bacteria-embedded constructs for therapeutic applications. Biofabrication enables the production of multi-dimensional structures inherently encapsulating living organisms, thus maintaining them alive and functional during the processing steps. Additionally, when compared to other encapsulation techniques (e.g., emulsification, freeze-drying, spray-drying), some methods have been revealed to entail a superior approach due to low energy costs, high loading efficiency, and the possibility of scaling up [5].

Among several well-established biofabrication methods, cell-electrospray, cell-electrospinning, and 3D bioprinting have emerged as attractive options to produce multi-scale bioconstructs, ranging from 0D (i.e., particles) to 1D (i.e., fibers), 2D (i.e., fiber meshes), and 3D (i.e., scaffolds), which can be used as novel bacteriotherapy devices [21,22,23]. Depending on the target site, such fabrication techniques allow scientists to work with several different materials. Furthermore, processing parameters can be tuned to obtain formulations suited for site-specific administration (e.g., proper pH and osmolarity for the respiratory tract or edible biomaterials with pH-responsive behavior for intestinal administration) [5]. Biofabrication of cell-loaded structures has largely been documented due to its wide use in various research fields, such as tissue engineering, regenerative medicine, drug screening and clinics [24,25,26]. Although in its infancy, the research on electrospinning, electrospray, and 3D bioprinting of bacterial cells is currently expanding [27,28,29,30]. Biomaterials have been used to provide the essential framework for encapsulating and safeguarding living organisms, ensuring their viability throughout the processing and delivery phases. In the context of GI disorders, for instance, bacterial cells, including probiotics, have demonstrated their potential as powerful therapeutic agents. However, their effectiveness is often impeded by challenges in surviving the harsh gastric conditions and colonizing the GI tract following oral administration [24]. Biomaterials play a pivotal role in overcoming these hurdles by encapsulating and shielding the bacteria, guarding them from the stomach harsh conditions, and facilitating their safe passage to the target site. By enhancing the retention and viability of these beneficial bacteria within the GI tract, biomaterials become essential partners for the success of bacteriotherapy, thereby improving its ability to effectively modulate intestinal flora [31].

### 2.1. The Electrospinning Process

The term “electrospinning” derives from “electrostatic spinning”, as this technique takes advantage of a Direct Current (DC) voltage in the range of several tens of kV to generate an electrostatic force to form fine fibers from a polymer solution or melt. A typical electrospinning apparatus consists of a high-voltage supply, a spinneret (e.g., a syringe needle), and a grounded collector with variable shape, such as a metal plate or a rotating mandrel [32,33,34]. The polymeric melt or solution is introduced in a capillary tube, usually with a vertical or horizontal setup, capable of pumping at a controlled rate. The high-voltage source injects a charge of a certain polarity into the polymer solution or melt, which is then accelerated toward the collector of opposite polarity when the repulsive electrical forces overcome the surface tension of the solution at the tip of the needle [33,35]. The polymer molecules must be long enough to avoid Rayleigh instability and breakage [36]. In proper electrical conditions, depending on the employed solution, the droplet becomes unstable, and a single fluid jet is drawn out from the so-called Taylor’s cone in an almost straight line. Due to the presence of many forces (i.e., Coulomb, electric, viscoelastic, surface tension, gravitational, and air drag forces) acting upon the jet, the onset of an unstable and rapid whipping of the jet can be observed, which occurs in the space between the tip and the collector. During the time of flight, the solvent evaporates, and a polymer fiber is formed and then collected upon a static or rotating collector [33,36].

Many parameters play a fundamental role during electrospinning, and all of them are relevant in determining the final properties of fibers in terms of morphology and structure. Electrospinning parameters include solution parameters (e.g., viscosity, conductivity, molecular weight, solvent vapor pressure, surface tension), process parameters (e.g., applied electric field, tip to collector distance, feed rate, inner diameter of the needle, shape of the collector), and ambient parameters (i.e., temperature, relative humidity, atmospheric pressure) [33,34,37,38].

#### Polymers for Bacterial Cell Electrospinning

A variety of polymers have been used to produce electrospun nanofibers, e.g., tissue engineering scaffolds, wound dressings, drug-delivery systems, and filtration membranes [39]. Natural polymers usually show higher biocompatibility, lower immunogenicity, and an increased similarity to natural tissues, overall promising better clinical functionality. However, natural polymers may undergo partial denaturation, and their characteristics are less reproducible, often depending on the extraction source [33]. Furthermore, natural polymers can show poor mechanical properties, making them unsuitable for a multitude of applications [34,40]. Typical natural polymers include collagen, chitosan, gelatin, casein, cellulose acetate, silk fibroin, chitin, and fibrinogen. Synthetic polymers, on the other hand, can be tailored to achieve finely tuned properties, such as mechanical properties and degradability. Polyglycolide (PGA), polylactide (PLA), poly-ε-caprolactone (PCL), polyurethanes (PU), poly(vinyl alcohol) (PVA), poly(ethylene oxide) (PEO), polyhydroxyalkanoates (PHAs), and many copolymers are among the most widely used synthetic polymers for electrospinning procedures [33,39]. In principle, it is possible to electrospin all polymers into nanofibers, as long as the molecular weight is sufficiently high and the solvent can be evaporated quickly enough [32]. However, the optimization process may prove challenging for many types of polymers. In fact, biopolymers are generally difficult to electrospin, likely due to material properties such as molecular weight, morphology, entanglement concentration, extensional viscosity, surface tension, vapor pressure, and a tendency to form gels. These parameters can affect the capacity of the solution to form an adequate Taylor’s cone at the tip of the needle, as well as achieve the adequate rheological characteristics that allow the formation of fibers during the flight of the jet toward the collector [41].

**Figure 2:**
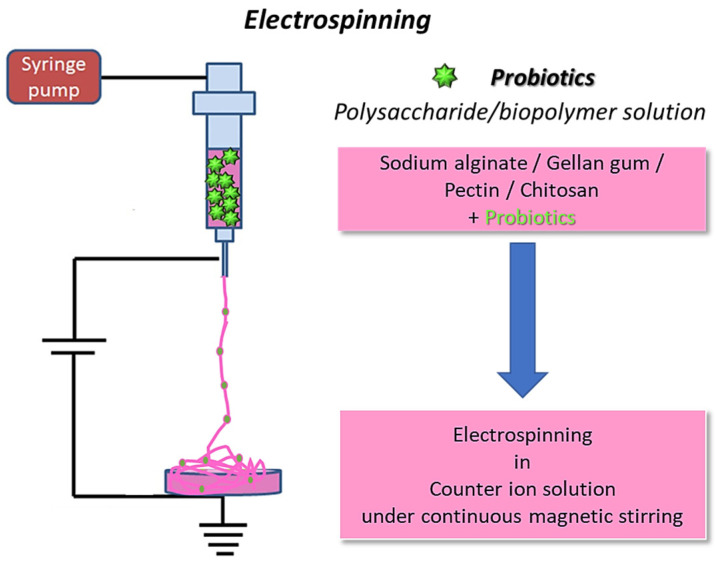
Schematization of bacterial cell electrospinning using biopolymers: the fibers are collected in a coagulation bath to allow polymer crosslinking (original image by the authors).

The fabrication process for electrospinning polymers with incorporated bacterial cells, schematically reported in Figure 2, encompasses several hydrogel-forming biomaterials, different crosslinking methods, and diverse incorporated bacterial cells.

Polysaccharides are extremely interesting materials for biomedical applications due to their biocompatibility, biodegradability, availability of chemical groups that can be functionalized, resemblance to the amorphous natural extracellular matrix (ECM), and ability to interact with cells. Chitosan, hyaluronic acid, sodium alginate (SA), cellulose, chitin, and dextran are among the most studied polysaccharides for biomedical applications [40]. Stijnman et al. investigated the possibility of electrospinning a wide variety of polysaccharides [41]. Necessary but insufficient conditions to achieve successful electrospinning are a high concentration in units of the overlap concentration and a weak tendency of shear thinning at shear rates below 1000 s^−1^ (the relevant regime for electrospinning). 

As reported by other authors, the degree of entanglement is a pivotal factor in ensuring fiber formation during electrospinning, and the entanglements must be present in the solution prior to solvent evaporation [42,43]. For polysaccharides, the entanglement-forming capacity strongly depends on the chain morphology; for instance, globular-like chains are less likely to form entanglements than random walk-like chains. Furthermore, if the polysaccharide solution shows a strong shear thinning behavior, this implies a lower number of entanglements and a decreased extensional viscosity at high shear rates. Basically, for polysaccharides to yield uniform fibers via electrospinning, it is necessary to achieve the condition C/C* > 10. Notably, earlier studies with poly(methyl methacrylate) (PMMA) [43] identified the onset of the semi-dilute entangled regime at C/C* ~ 3 and the formation of uniform fibers (regardless of the molecular weight) for C/C* > 6. This implies that polysaccharides generally require higher concentrations to achieve fiber formation. However, increasing the concentration of the polysaccharides that are not spinnable is not always possible due to different factors, such as lack of solubility, excessive viscosity, or the tendency to form a gel. Moreover, many of the charged polysaccharide solutions maintain a structured, weakly gelled character and present yield stress; C/C* is the ratio between the concentration of the polymer in the solution (C) and the overlap concentration (C*), i.e., the concentration that marks the transition from the dilute regime to the semi-dilute unentangled regime and represents the onset of chain interactions. Due to the high surface tension and strong entanglement of hydrogel polysaccharides, the addition of water-soluble polymers, such as PVA and poly(ethylene oxide) (PEO) as carrier materials is one of the most studied methods for electrospinning [44]. 

**Figure 3:**
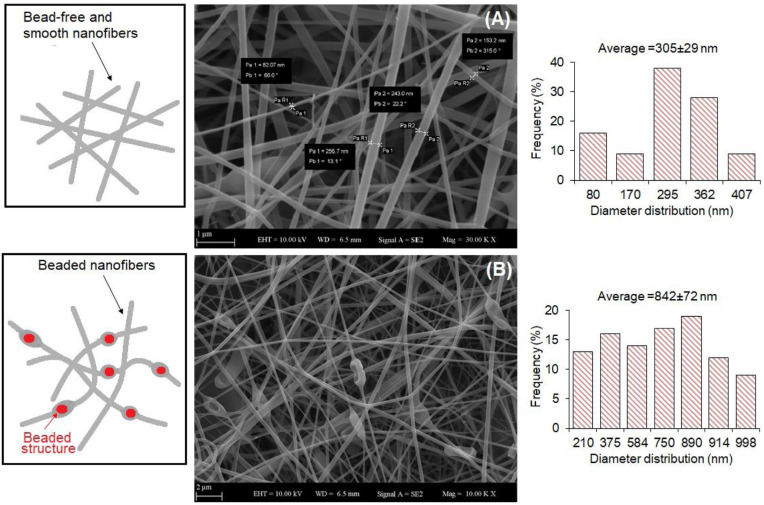
Example of produced electrospun SA/PVA fibers: (**A**) empty (average diameter: 305 ± 29 nm), and (**B**) loaded with *Lactobacillus paracasei* strain (average diameter: 842 ± 72 nm, with bead formation), which have a viability rate of 85.87% ± 0.78% after the electrospinning process. Reprinted with permission [48]; © 2020 Elsevier.

Fareed et al. investigated the gut fate of probiotic-loaded nanofibers. Specifically, *Lactobacillus acidophilus* strains were loaded in a mixture of Arabic gum and PVA and were electrospun into nanofibers [45]. The fibrous system displayed a good encapsulation capability, improving the tolerance to simulated gastric juice (SGJ) and successfully protecting the microorganisms against a harsh environment. The studies on the fabrication of probiotic-laden SA fibers via electrospinning have so far demonstrated the feasibility of the process. Several *Lactobacillus* strains, such as *Lactobacillus reuteri* [46], *Lactobacillus plantarum* [47], and *Lactobacillus paracasei* [48], were successfully encapsulated in SA-based nanofibers, as shown Figure 3. 

For instance, Ceylan et al. successfully fabricated PVA/SA nanofibers with an average diameter of 583 nm [46]. The viability of probiotics was successfully maintained during the electrospinning procedure, as 83% of encapsulated bacteria were alive afterward. Reportedly, Lactic Acid Bacteria (LAB) can produce an acidic environment by converting sugar to lactic acid, thus inhibiting the growth of harmful bacteria. In fact, the authors were able to extend the cold storage of fish fillets by enwrapping them in probiotic-loaded nanofibers, which effectively reduced the growth of psychrophilic bacteria, i.e., cold-loving bacteria [49]. Feng et al. produced core–shell nanofibers using a PVA-SA blend for the shell and a probiotic-loaded PVA core [47]. The nanofibers (270 ± 64 nm) showed a beaded core–shell structure and were able to protect probiotics both during the fabrication and after exposure to simulated gastric juice and simulated intestinal juice (SIJ). Xu et al. developed a batch of PVA/Pectin (PEC) electrospun fiber meshes embedding the *Lactobacillus rhamnosus.* Based on the composition of the polymer, they obtained meshes with different average diameters between 112.30 ± 78.10 nm and 149.89 ± 25.66 nm. Although the mechanical properties of the fiber meshes were not assessed, the authors demonstrated the high viability of the probiotics in a time span of 21 days [30].

Electrospinning has proven effective in encapsulating significant bacterial loads within fibers at the laboratory scale, showing promise for applications such as drug delivery, wound dressings, agriculture, and food industry products [50]. However, the field of bacterial electrospinning still faces several challenges affecting cell viability, including insufficient focus on parameter-based studies in the current literature and reproducibility issues [51]. The high voltage used is generally considered a worrisome condition for bacterial cell survival during electrospinning, as electric and mechanical forces (e.g., those occurring during solvent evaporation and jet stretching) might induce cell break. However, many studies have shown that bacteria can survive the typical voltages applied in this process (e.g., 15 kV), as the electric current within the fiber usually remains low due to the high resistivity of the polymer [50]. Salalha et al. studied the viability of *Escherichia coli* and *Staphylococcus albus* in electrospun PVA fibers before and after the electrospinning process and found that the viability was species-dependent; in fact, immediately after electrospinning, the viability of *E. coli* was 19%, whereas *S. albus* resulted 100%. It was also demonstrated that the viability of *E coli* was affected by its growth phase (i.e., exponential versus stationary) and by the growth medium used. Adding 5% glycerol increased bacterial viability to 48%, imputing this positive effect to a protective role of glycerol during the rapid dehydration (~10 ms) experienced by the fiber solution during the process [52]. Therefore, it was concluded that the fast evaporation of the solvent played a key role in *E. coli* cell survival. In a subsequent study, 10 types of *Lactobacilli* were incorporated within electrospun PEO fibers, showing viability reduction within 0–3 log CFU/mg, depending on the species. These authors correlated viability with the hydrophobicity and extreme length of lactic acid bacteria, whereas the electrospinning configuration (i.e., horizontal or vertical) did not affect the bacterial viability [53].

Surely, water represents the best solvent for entrapping living matter inside polymeric structures, including bacteria. However, the final device properties could sometimes be better tuned to the specific application by using polymers that do not dissolve in water or need solvent/water mixtures, which could be harmful to bacteria. Addressing these challenges requires formulating environmentally friendly green solvent systems and conducting thorough parametric studies, considering the shear stresses involved and minimizing variations across batches [51,54]. A summary of the main results from works on electrospinning probiotics-loaded polymers is reported in Table 1.

### 2.2. Electrospraying

Electrospraying is an electrodynamic technique like electrospinning, which allows the fabrication of nano- and micro-particles in the form of solid spheres or capsules [34,40]. The apparatus required to carry out an electrospray procedure is like that employed for electrospinning. During the electrospray process, a Taylor’s cone is formed at the tip of the needle, where several types of forces act, i.e., an electrostatic force, surface tension, and gravity [55]. Here, the surface tension is normally lower compared to that experienced in electrospinning conditions, and the electrostatic force causes the deformation of emitting droplets to spherically shaped jetting beads. Therefore, a spray is formed instead of filaments, leading to the formation of small particles upon solvent evaporation. 

**Figure 4:**
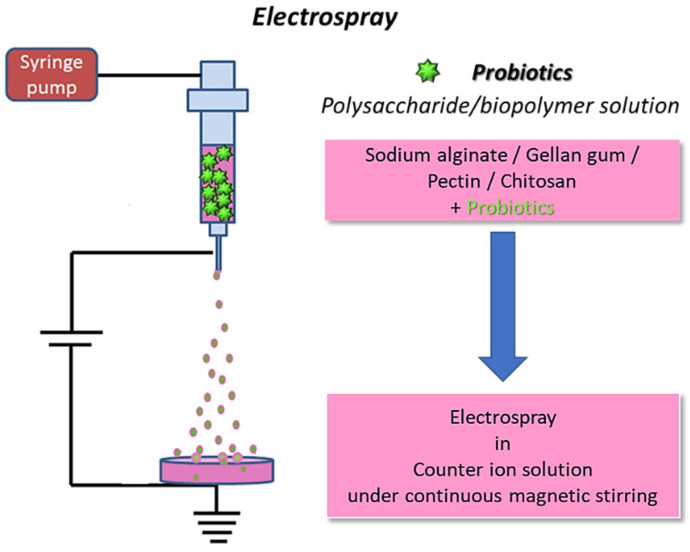
Schematics for electrospraying of biopolymers loaded with probiotics: the fibers are collected in a coagulation bath to allow for polymer crosslinking (original image by the authors).

Electrospraying allows the production of nano- or micro-structures with a large surface-to-volume ratio; good porosity; high encapsulation efficiency; and possible protection of the active compound from possible adverse factors, such as the pH, enzymes, water, light, and oxygen both during the fabrication and delivery. Furthermore, particles produced through electrospray are small and uniform and allow for a great control over the delivery of the encapsulated agent to be achieved [56]. Although less popular than electrospinning, electrospraying has also been employed for microencapsulation of probiotics (Figure 4). 

The current literature on probiotic encapsulation via electrospraying is still sparse, but many promising results have been shown. High survival rates of probiotic cells against the high voltage and stress associated with electrospraying have been documented. Differently from electrospinning, watery solutions of polysaccharides are easily addressed via electrospraying; thus, these biopolymers are often used due to their low cost, suitable chemistry, high biocompatibility, biodegradability, and favorable biological activity [57]. As an example, SA is a popular biopolymer used for electrospraying and has been used to encapsulate different bacterial strains, such as *Lactobacillus acidophilus* [58,59], *Lactobacillus plantarum* [60,61,62], *Bifidobacterium lactis* [62], or a mixture of these [63]. Several authors reported the successful fabrication of probiotic-laden microparticles or microcapsules with very high cell loads and diameters (e.g., typically in the range of hundreds of microns) suitable for delivery, e.g., via the GI route.

Such particles are usually tested in simulated body fluids, like SGJ and SIJ, to assess the ability of these delivery systems to support bacterial viability in harsh conditions and guarantee survival and arrival at the target site. Because the GI tract is the most common target for probiotic delivery, many of these studies focused on the improvement in probiotic survival by means of coating the SA particles with a shell of a protective material, such as pectin [60], zein [58], and chitosan [61,62]. These studies demonstrated the ability of the coating materials to successfully protect probiotics during their residence in simulated gastric and/or intestinal environments. Furthermore, the presence of a second layer can influence the release rate of the encapsulated probiotics and any additional cargo, e.g., inulin and resistant starch [62]. 

**Figure 5:**
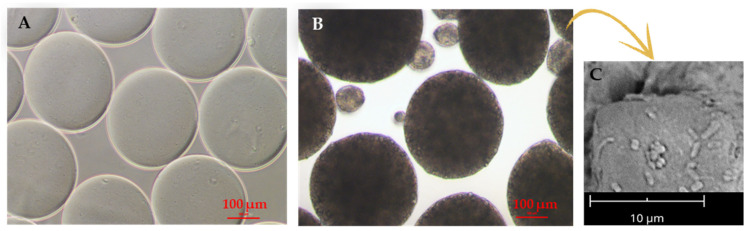
Electrosprayed SA microparticles used for probiotic encapsulation. (**A**,**B**) Optical micrographs of (**A**) empty and (**B**) probiotic-laden microparticles, as produced in the crosslinking bath; scale bar is 100 µm. (**C**) Scanning electron micrograph of microparticles showing probiotics at the surface; 5000× magnification at 5 kV, scale bar 10 µm. Adapted from [54] and reproduced under the terms and conditions of the Creative Commons Attribution (CC BY-NC-ND) license.

In a different vein, Saber Amiri et al. achieved success in electrospraying *Lactobacillus acidophilus* LA-5 into whey protein isolate (WPI)/lactose spherical nanocapsules [29]. Notably, after 28 days of storage at both 4 °C and 20 °C, a non-significant reduction in the viability of encapsulated bacterial cells was observed, highlighting the stability of the formulation [29]. Addressing chronic otitis media, Cecchini et al. explored the use of probiotic-laden, electrospun microcapsules against the *Escherichia coli* pathogen [63]. The microcapsules preserved both the viability and functionality of entrapped beneficial bacteria, exhibiting remarkable antimicrobial activity [63] (Figure 5).

Although electrospraying has demonstrated success in encapsulating active compounds, certain challenges persist. The technique excels in producing nano- and micro-structures with a large surface-to-volume ratio, high encapsulation efficiency, and protection of active compounds from adverse factors. Many of the limitations to bacterial cell viability described for electrospinning have a milder effect when using electrospraying because the mechanical (i.e., solvent evaporation and filament stretching) and electrical forces (employed electric field) are lower than those experienced in electrospinning. Nevertheless, achieving precise control over particle size and morphology remains a crucial goal, given their significant impact on the sustained release properties of bioactive compounds [64]. 

Additionally, using electrospraying for large-scale production encounters obstacles due to its inherently low-volume processing. To overcome these challenges, ongoing research is focused on enhancing production efficiency and exploring innovative approaches like multiple jet and multi-nozzle methods [65]. Table 2 presents an overview of the literature on probiotics-loaded polymers that were successfully electrosprayed.

### 2.3. The 3D (Bio)Printing Process

**Figure 6:**
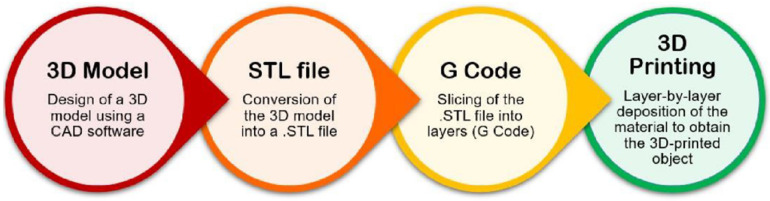
Flow diagram representing the main steps of the 3D printing process (original image by the authors).

Three-dimensional printing is an additive manufacturing (AM) technology. It is a widely researched, highly efficient fabrication technique that finds application in many fields, such as regenerative medicine, drug delivery, materials science, aerospace, automotive, art, construction, toys, food industry, and sports accessories [66,67]. Also, 3D printing enables the construction of simple to complex structures by adding the material layer-by-layer starting from a 3D computer-aided design (CAD). A schematization of the main steps of the 3D printing process is reported in Figure 6. 

AM offers many advantages over traditional subtractive manufacturing techniques, including expediency, design independence, and reduced material waste. Ink-based methods, including extrusion- and inkjet-based 3D printing techniques, use a robotically controlled printhead for printing and represent the gold standard for shear thinning polymers [68]. Over the years, AM and extrusion-based 3D bioprinting has been used as a powerful method to fabricate tissue and organ-like structures, with increasing popularity in the fields of tissue engineering and regenerative medicine. 

The printing materials are often hydrogel-based inks because of their biocompatibility and inherent characteristics similar to those of natural tissues. Inks for biofabrication can be classified as bioinks or biomaterial inks [69]. Bioinks are cell-laden formulations, mainly based on aqueous and hydrogel precursor media, in which living organisms (e.g., human and animal cells, bacteria, fungi, bioactive molecules, or a combination thereof) are dispersed. To provide an adequate niche for cells to thrive, such hydrogels are typically characterized by low elastic moduli and a biochemical composition suitable for cell-driven renewal. On the other side, biomaterial inks are not directly formulated with cells but can be printed and subsequently seeded with cells, thus allowing working with a wider window of processing parameters (e.g., higher pressure and temperature and the use of organic solvents) during the printing phase without damaging the cells [69,70,71,72,73,74].

#### Bioprinting of Probiotic-Loaded Constructs

Hydrogel precursors have been extensively exploited to formulate inks for 3D extrusion-based bioprinting [70]. Natural hydrogels, such as SA, collagen, gelatin, hyaluronic acid, agarose, or silk, have been widely used as bioink components due to their similarities to the native extracellular matrix (ECM), while synthetic materials like polyethylene glycol (PEG) and poloxamers are often selected for their tunable mechanical properties and lower batch to batch variability [31,70,75].

**Figure 7:**
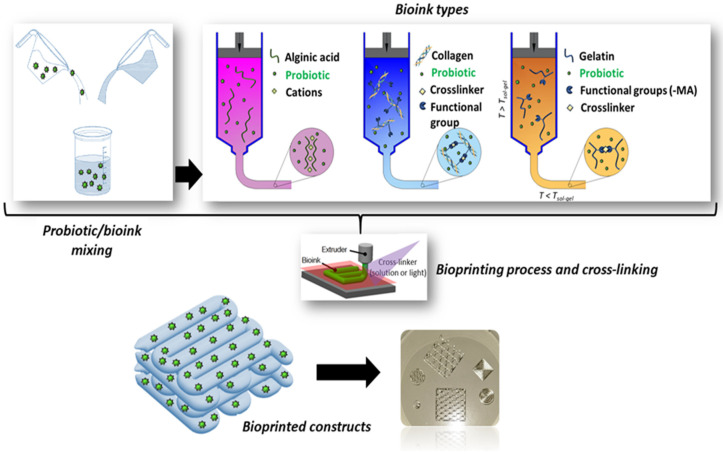
Schematic of the crosslinking mechanisms and 3D bioprinting process used to fabricate probiotics-loaded constructs using different biopolymers, e.g., SA, collagen, and gelatin (original image by the authors).

Hydrogels are 3D crosslinked networks of hydrophilic polymer chains that can retain water contents several times their own weight while maintaining their structure [67]. Hydrogels are soft and moist, can recover under the action of external forces, and have large specific surface areas. A schematic of the crosslinking and fabrication processes is reported in Figure 7.

According to the crosslinking method, hydrogels can be categorized into physical hydrogels and chemical hydrogels. Physical crosslinking occurs through the formation of physical bonds (e.g., electrostatic interactions, hydrogen bonds, and pH and/or thermal responses) and is favored for bio-extrusion due to the mild treatment conditions and reversible gelation. Nevertheless, the physical hydrogels are inherently weak and unstable, resulting in being disadvantageous for long-term in vitro cultures and/or in vivo [31,75]. On the other hand, chemical hydrogels have three-dimensional networked structures formed by covalent bonding and are stable for longer periods than physically crosslinked hydrogels [66]. Additionally, the mechanical strength of chemical hydrogels can be suitably tuned by controlling the crosslinking density. Among chemical-based gelation processes, UV crosslinking is the most commonly applied for the bio-extrusion of pre-gel formulations. However, exposure to UV light and free radicals can decrease the cell viability of the bioink [75,76]. For reliable 3D bioprinting, hydrogel constructs should be biocompatible and provide a non-toxic and promotive microenvironment for various vital functions of seeded or embedded cells. Additionally, the bio-constructs should not cause adverse immune responses or even elicit a beneficial immunomodulatory activity when used for in vivo investigations [31,75].

SA has been largely employed to prepare biomaterials and bioinks for extrusion 3D bioprinting of hydrogel-based bio-constructs for different biomedical applications. SA, as a natural biopolymer, is safe, biocompatible, and biodegradable. SA can physically crosslink in the presence of nontoxic divalent cations and under benign conditions, making it attractive for cell encapsulation. In addition, the favorable functionality of SA (α-L-guluronic (G) and 1-4-β-D-mannuronic acid (M) monomers), which allows for structural modifications, together with its hydrophilic nature, enables the preparation of new ink formulations suitable for extrusion bioprinting [77]. The use of SA-based biomaterials and bioinks for advanced medical applications is still limited due to their poor mechanical stability (i.e., low stiffness, unstable swelling, and degradation behavior) and bioactivity. Therefore, to enhance the extrusion 3D bioprinting of SA, it is often chemically modified and/or functionalized with several polymers and nanomaterials [77,78].

Mallick et al. used 3D bioprinting to encapsulate probiotics (i.e., *Lactobacillus rhamnosus*) for gut delivery [5]. The bacteria were loaded into SA-gelatin-based ink and printed into capsule form. The probiotics were uniformly distributed. The probiotics were uniformly distributed inside the capsules and remained viable for up to 7 days when exposed to GI fluidic conditions. Three-dimensional printing has also been exploited for the fabrication of artificial biofilms. It was reported that probiotic biofilms could be 3D-printed onto various biomedical implant surfaces to prevent device-associated infections caused by pathogenic bacteria [79]. However, current literature mostly focuses on bacterial printing for other applications (e.g., the detoxification of wastewaters, the preparation of model biofilms, and biocatalysis), and the fabrication of 3D-printed platforms for bacteriotherapy requires further investigation. Nevertheless, the findings related to the manufacturing of 3D-printed biofilms for different applications could provide a solid base for the development of probiotic biofilms. 

**Figure 8:**
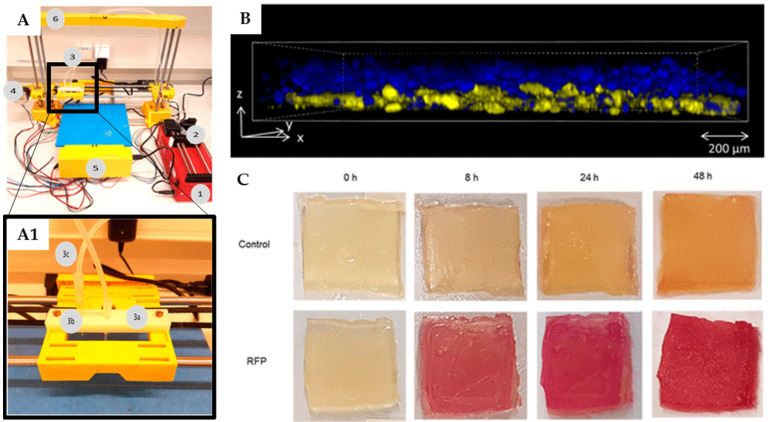
Bacterial biofilm 3D bioprinting system: (**A**) Overview of the bioprinter components. 1: syringe pump, 2: syringe filled with bioink, 3: extruder holder, 4: one of three step-motors for positioning, 5: breadboard and hardware of the printer, 6: frame of the printer. (**A1**) Detailed view of the modified extruder; 3a: active pipet tip, 3b: secondary pipet tip for layering materials, 3c: tubing system. (**B**) Internal structure via scanning confocal laser microscopy of printed layers modified strains of *E. coli* expressing 2 different fluorescent proteins after 24 h of incubation: the bottom layer contained 81% ± 5% blue fluorescent cells, while the top layer contained 93% ± 5% yellow cells. (**C**) Metabolic activity of *E. coli* with and without a rhamnose-inducible red fluorescent protein (RFP) plasmid printed within alginate gels onto a substrate containing rhamnose; color change as observed over time. Adapted from [80], © 2017 American Chemical Society licensed under CC-BY-NC-ND.

In 2017, Lehner et al. used a modified commercial 3D printer to fabricate artificial biofilms (Figure 8) [80]. A liquid mixture of bacteria (i.e., *Escherichia coli*) and SA was used as a bioink and printed upon a calcium chloride-treated printing surface, thereby rapidly solidifying into a gel. The best concentrations of alginate and molarity of calcium chloride were reported to be 2.5 *w*/*v* and 1 M, respectively. The system was able to print details of sub-millimetric resolution and deposit bioink directly on top of previously printed material to create multilayered structures. Moreover, different fluorescent proteins produced by engineered two *E coli* strains, encapsulated during the production of a bi-layered structure, showed a substantial separation of the two bacteria, thus indicating minimal mixing. Biofilms were also tested for bacterial viability, indicating strong bacterial growth during the first 24 h after gel production and maintenance of a fairly constant number of colony-forming units for up to 48 h. Additionally, bacterial cells in these conditions exhibited a strong metabolic activity, suggesting that the printed bioink is able to support the production of bacterially made materials over short periods of time [80]. 

Further advancements in this direction were made by Schmieden et al. in 2018 [81]. The authors used commercially available toy components to build a cheap 3D printing system capable of printing features with a line width resolution of approximately 2 mm. The bioink consisted of a mixture of bacteria, liquid growth medium, and alginate, while calcium-impregnated agar plates served as printing substrates. The alginate molecules of the bioink complexed with the calcium ions of the printing substrate, allowing for the polymerization and the formation of a gel. This system was also capable of printing multilayered structures. However, the line width increased fractionally with each layer added (~14%), likely due to the decrease in calcium concentration along the *z*-axis, which in turn caused a delayed gelation of successive layers. In fact, the gelation time spanned from seconds (for the first layer) to 5 min (for the fifth layer). Nonetheless, it was shown that the alginate hydrogel allowed free diffusion from the substrate to the upper layers. Further tests confirmed the ability of these gels to maintain the bacteria furnished with nutrients and inducers diffusing from the printing substrate. Fluorescence and viability experiments demonstrated the capability of bacteria to remain alive and useful for roughly 1 week after printing. The main limiting factors for bacterial survival were likely nutrient depletion, accumulation of waste products, and drying of the gels and plates. However, the authors suggested that drying could be delayed by sealing the plates with plastic wrap and supplementation of water. In order to keep bacteria alive for a longer time, fresh nutrients could be supplied, and waste products could be removed by submerging the printed gels in a growth medium supplied with calcium chloride to avoid gel dissolution [81]. Alginate-based hydrogels are popularly used for 3D printing technologies due to their excellent printability and biocompatibility, low cost, low toxicity, and rapid gelation. However, due to the low viscosity and the inherently weak mechanical performances that accompany physically crosslinked hydrogels, the preparation of complex alginate structures with high fidelity might be difficult. In order to fix this issue, a good approach consists of combining alginate with a supporting material [82].

For instance, in 2020, Freyman et al. loaded their alginate-based bioink with cellulose to provide stability to the ink and aid in flow during printing [83]. In this case, the 3D printed structure was exposed to a calcium chloride solution after printing, and ionotropic gelation occurred. The resulting structure was mechanically stable and flexible, and a live/dead assay confirmed the viability of entrapped bacteria. Further tests indicated the ability of bacteria to grow and perform the desired functions inside the 3D-printed matrix [83]. Because of their antimicrobial properties, cationic polymers such as chitosan should be avoided in bacteriotherapy applications. Hyaluronic acid (HA) can be used in combination with alginate to form a double-network hydrogel with good adhesion properties. Gelatin has widely been used in combination with alginate to prepare hydrogel platforms for the expansion of stem cells. Among synthetic polymers, instead, poly(ethylene glycol) proved to be a good copolymer for wound-healing applications [82]. 

A different approach was followed by Schaffner et al., who created a multi-material hydrogel for bacterial 3D printing [84]. They developed a new biocompatible living ink called “Flink” composed of HA, κ-carrageenan (κ-CA), and fumed silica (FS). Rheological studies allowed the optimization of the bioink, which showed good viscoelastic properties (i.e., shear thinning with fast structure recovery) for direct ink writing while ensuring a high survival rate for bacteria. A 1:1:1 ratio of the HA/κ-CA/FS constituents was identified as the optimal composition ratio, while the viscosity and elasticity of the hydrogel increased when the overall concentration was brought from 3 w% to 6 w% and 9 w%. However, since the ideal viscosity ultimately depends on the final application of the hydrogel, the concentration should be tuned accordingly. Another approach involved the substitution of HA with chemically modified glycidyl methacrylate HA (GMHA). This replacement did not significantly alter the viscosity and allowed the hydrogel to be UV-crosslinked at low exposure doses and innocuous wavelengths to form a water-insoluble hydrogel. Flink-based hydrogels were cytocompatible with regard to loaded bacteria, and the presence of radicals during UV exposure was not harmful. Moreover, additional tests were performed to assess the usefulness of bacteria by investigating the ability of *P. putida* strains to degrade phenol into biomass as well as the ability of *X. xylinum* to produce bacterial cellulose when exposed to oxygen in a culture medium. Both tests confirmed that bacteria retained their metabolic activity and were able to grow and proliferate when embedded in Flink hydrogels, which, in turn, have the advantage of providing a predesigned environment with a defined and complex shape [84].

In an innovative stride, Kryser and colleagues explored the potential of bioprinting for sustained delivery of *Lactobacillus crispatus* in the female reproductive tract [28]. Probiotics-loaded 3D printed gelatin/alginate scaffolds, formulated in a 10:2 weight ratio, displayed fine printing resolution, mechanical integrity over 28 days, and the remarkable capability to sustain the viability of probiotics. The controlled release suggested a potential advantage in outcompeting pathogens, positioning this formulation as a promising prophylactic measure against dysbiosis in the delicate environment of the vaginal microbiome [28]. Three-dimensional printing allowed the microencapsulation of *Lactobacillus rhamnosus* CNCM I-4036 (Lr) inside, generally recognized as safe (GRAS) proteins for oral delivery formulations intended to reach the GI tract [85]. The bacteria were pre-encapsulated in microparticles (MP) of 12.3 ± 4.1 μm to preserve cell viability and thereafter 3D-printed into final formulations of 15 × 8 × 3.2 mm^3^ oval-shaped size and ~370 mg weight. After the 3D-printing process, bacterial viability remained like that detected in MP-Lr protected bacteria, showing a statistically significant difference (i.e., 0.52 log reduction with respect to 3.05 log reduction in the non-encapsulated probiotics). Extrusion bioprinting was used by Li and coworkers to immobilize the heterotrophic bacterium *Oceanimonas sp.* XH2, capable of removing ammonia, in a dually crosslinked PEG-diacrylate (DA)-alginate-PVA-nanoclay (PAPN) bioink. The bacteria remained viable after processing, and the PAPN printed structures could remove up to 96.2% ± 1.3% ammonia in 12 h. Moreover, the authors concluded that the bioink design remained a critical challenge due to the difficulty in creating a durable and bio-friendly material [86].

Moreover, 3D-printed microcontainers have emerged as promising solutions for improving colon-targeted probiotic delivery [14]. Dual-compartment microcontainers (DCMCs) were fabricated via 3D printing technology, employing a pH-sensitive polymeric formulation based on Eudragit^®^ L100 and S100, and enriched with probiotics. In vitro assessments involving *Lactobacillus rhamnosus* GG (LGG) demonstrated the effective release and survival of probiotics within the microcontainers. Progressing to in vivo studies, the microcontainers were used for the delivery of LGG and *Escherichia coli*; for both strains, the DCMSs exhibited a remarkable ability to deliver viable probiotics, showcasing significant adherence to the colonic mucosa [14]. Despite the huge progress in 3D bioprinting of probiotic-laden constructs, persistent challenges still impact both the printing process and the employed bioinks. The delicate balance required in the selection of hydrogel precursors, considering printability and stability, reveals inherent limitations in mechanical performance. While promising strategies involve the incorporation of supporting materials and the development of multi-material hydrogels, further optimization is essential [74].

**Figure 9:**
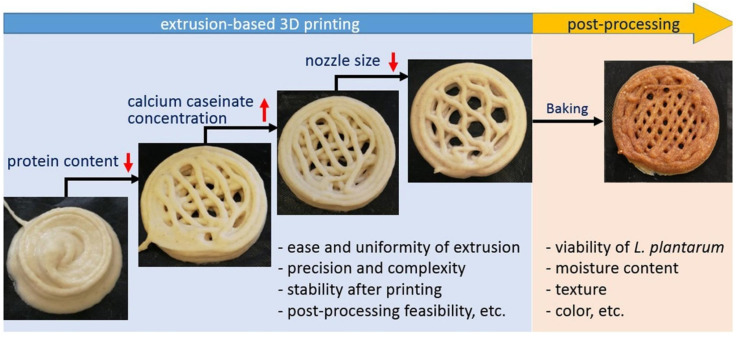
Schematics showing extrusion-based 3D bioprinting of cereal structures containing probiotics for intestinal delivery [88]. Reproduced under the terms and conditions of the Creative Commons Attribution (CC BY-NC-ND) license.

Recently, 3D printing has also become popular in the food industry (Figure 9). The incorporation of *Bifidobacterium animalis subsp. Lactis* into 3D-printed mashed potatoes was attempted by Liu et al., who investigated the rheology of the formulations [87]. These authors focused on the facts affecting cell viability and found a slightly unfavorable effect on probiotic viability exerted by extrusion within a small nozzle diameter (0.6 mm) and a largely unfavorable effect exerted by temperature (55 °C) and time (45 min) when the MP was held in a heating nozzle barrel. Yield stress and elastic modulus G′ were critical properties needed to ensure proper performance of MP. An interesting study by Zhang et al. has additionally suggested the bioprinting of cereal-based food structures containing probiotics, which were interestingly able to survive baking [88].

Moving beyond material considerations, current 3D bioprinters grapple with limitations in speed, scalability, and versatility when handling diverse living microorganisms-based inks. Overall, 3D bioprinting has emerged as a safe technique to process probiotics, even though some authors have proposed strategies to further reduce the effect of the process, such as pre-encapsulating bacteria in microparticles to be loaded into the ink before printing [85,87]. The need for high precision and survival rates, coupled with the challenge of precise cellular patterning at limited resolutions, underscores the importance of tailoring bioinks to the accuracy requirements of extrusion-based bioprinting technologies [74]. Looking forward, the future of 3D bioprinting hinges on overcoming these challenges. Developing affordable, versatile, and up-scalable bioprinters capable of efficiently handling various bioinks is crucial for advancing this technique. As progress is unfolding in 3D modeling software applications and bioprinting technology, addressing existing challenges in 3D bioprinting will pave the way for its widespread application across diverse biomedical fields [75]. An overview of 3D-printed probiotic-loaded constructs is reported in Table 3.

## 3. Delivery Systems Based on Probiotics

Inherently, the biomaterials used for biofabrication processes can be biodegradable or non-biodegradable at the target site. After crosslinking, the biopolymers can be stable in aqueous fluids for a certain period. In many cases, hydrogels undergo an initial swelling, which may be followed by biodegradation. The high water content of synthetic hydrogels and polysaccharides makes them ideal for entrapping, protecting, and helping to keep viable living cells, such as probiotics. These cells are supposed to be delivered to the target tissue and released to populate the site-specific microbiome by using suitable polymeric carriers. For instance, a 3D-bioprinted scaffold containing the vaginal lactobacillus *L. crispatus* was recently developed with the aim of controlling bacterial vaginosis, a common disorder in women of reproductive age linked to low levels of lactobacilli in the vagina and concomitant overgrowth of potential pathogens [28]. Different weight-to-volume ratios of gelatin and alginate and crosslinking reagents were investigated to identify the best printing parameters and scaffold stability. The optimized scaffolds demonstrated sustained release and proliferation of encapsulated bacteria over 28 days (at least 1 × 10^8^ to 4 × 10^8^ CFU of daily *L. crispatus* per mg of scaffold) without a negative effect on the viability of vaginal epithelial cells. This study provides in vitro evidence that 3D-bioprinted scaffolds may represent an innovative strategy for lactobacilli delivery with the aim of restoring the vaginal ecosystem following microbiological disturbances.

At least part of the therapeutic potential of live probiotics relies on their ability to produce a wide array of antimicrobial substances, which includes organic acids, fatty acids, hydrogen peroxide, carbon dioxide, bacteriocins, or bacteriocin-like substances [89]. Thus, an emerging alternative to the use of live bacteria as anti-infective biotherapeutics is the use of active components or metabolites thereof, also referred to as post-biotics [90]. Although probiotics are “Generally Recognized as Safe (GRAS)”, in certain circumstances, postbiotics may ensure a higher margin of safety, e.g., in immunocompromised subjects or pediatric patients, other than being easily produced and stored [91]. Besides their direct antimicrobial properties, anti-inflammatory, anti-proliferative, and immunomodulating activities have also been attributed to postbiotics, thus suggesting their use in a wide range of pathological disorders [90,92]. Although postbiotics have demonstrated promising performance in several in vitro systems, there are still main challenges to be resolved for their in vivo delivery, which must stimulate intensive research in this field [93]. Delivery systems based on lipids or polysaccharides are the most frequently applied for encapsulation of the therapeutic compounds [93,94,95]. Few examples exist of such systems applied to the delivery and release of postbiotics. For instance, liposome encapsulation of bacteriocin-based postbiotics has been investigated in terms of the bio-preservation of food, demonstrating the ability to enhance the stability of the bioactive molecules, to reduce unwanted interaction with food components, and to increase antimicrobial activity [96].

Recently, prebiotics have been used as carriers for the fabrication of microcapsules containing the post-biotic product indole-3-propionic acid (IPA) via microfluidic electrospray technology [97]. Prebiotics are non-digestible dietary carbohydrates, such as alginate, chitosan, and inulin, utilized as organic nutrients by the gut microflora, while IPA is a tryptophan metabolite produced by gut bacteria, demonstrated to inhibit gut dysbiosis [98]. By exploiting a possible synergistic effect between post-biotics and prebiotics, the purpose of the authors was to treat colitis, a disorder with multifactorial etiology recently associated with dysbiosis of gut microbiota [99]. Successful encapsulation of IPA into prebiotic microcapsules composed of alginate and resistant starch was achieved. A second coating layer of the microcapsules made of chitosan was applied to avoid IPA leakage during passage through the acid environment of the stomach, meanwhile ensuring its release at the neutral pH of the lower GI tract. In vivo experiments demonstrated that, compared to the administration of a sole prebiotic or post-biotic, mice treated with the combined ingredients showed a significantly lower trend of disease activity index and an increase in short-chain-fatty-acid-producing beneficial bacteria, such as *Faecalibacterium* and *Roseburia*, with respect to controls, thus suggesting that the employed strategy could also have the potential to correct dysbiosis.

Another interesting strategy for post-biotic delivery at the site of infection was investigated by Ming and coworkers [100]. The authors encapsulated live *Lactobacillus reuteri*, a known probiotic, into hydrogel microspheres via emulsion polymerization and further immobilized it in a hydrogel network via covalent crosslinking of methacrylate-modified hyaluronic acid. The resulting scaffolds allowed the sustained release of bacterial-derived antibacterial substances such as organic acid or reuterin, a potent antimicrobial agent active against both Gram-positive and Gram-negative bacteria [100]. In contrast, the scaffolds prevented the encapsulated bacteria from escaping into the surrounding environment, a property that, following in vivo administration, could avoid potential risks and protect lactobacilli from host-immune system attack. The efficacy of the hydrogel scaffolds was demonstrated in vitro against common wound pathogens and in vivo by using a full-thickness cutaneous wound infection model with *Staphylococcus aureus*. The wounds of mice treated with the scaffolds exhibited an accelerated closure time, while inflammatory cell infiltration was reduced, and collagen deposition was enhanced, indicating that *S. aureus* infection was efficiently controlled. Intriguingly, post-biotic formulations are emerging as promising candidates for cosmetic products, offering advantages such as the absence of bacteremia and fungemia risks, besides inherent stability during industrial processes and shelf life [101]. 

A notable instance is found in the recent study by Ashoori et al. [102], which delved into the characterization and application of topical formulations, each containing 1 g of probiotic lysates from *L. reuteri*, *L. fermentum*, and *B. subtilis sp.* in 100 g of chitosan nanogel (1% *w*/*w*) and designed for in vivo wound healing. The capsules, characterized by spherical and uniform structures ranging 10–50 nm, exhibited the best physical stability. Importantly, all probiotic lysate formulations demonstrated advantages in the wound-healing process, highlighting the effectiveness of chitosan nanogels as carriers for post-biotics in cosmetic formulations [102]. In conclusion, these diverse delivery strategies underscore the evolving landscape of probiotics and postbiotics in biomedical applications, offering potential solutions to challenges in stability, safety, and targeted delivery. Further research into these innovative approaches promises advancements in biotherapeutics and personalized medicine.

## 4. Conclusions and Future Perspectives

**Figure 10:**
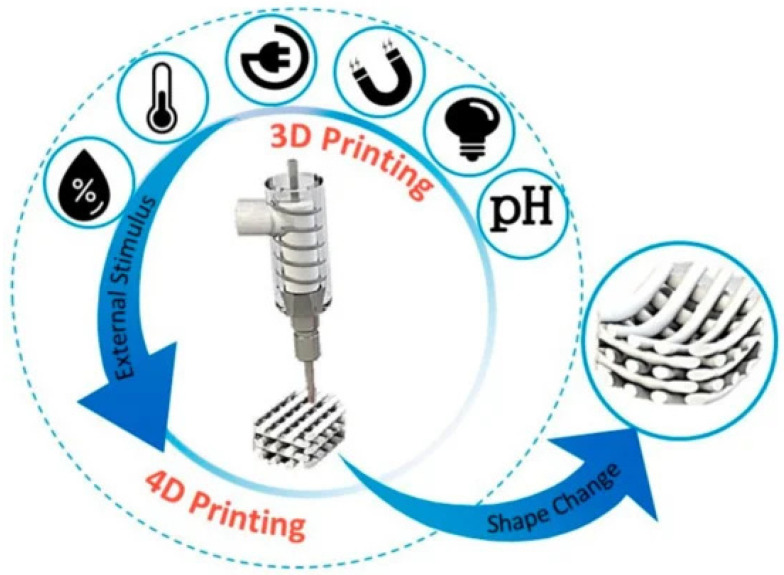
Schematic representing the diverse stimuli contributing to 4D (bio)printing, i.e., using biomaterials able to respond with a change of one of their morpho-physico-chemical properties (e.g., shape, size, color, rheology, and stiffness/elasticity) to a local stimulus (e.g., pH, light, magnetism, electricity, temperature, and humidity) [103]. Reproduced under the terms and conditions of the Creative Commons Attribution (CC BY-NC-ND) license.

Bacteriotherapy is emerging as a promising technique to prevent and/or treat dysbiosis of a variety of body districts (e.g., respiratory tract, skin, ear, among others). Additive manufacturing technologies have been only recently considered as powerful tools to fabricate bacterial cell-loaded devices to locally deploy probiotics. Electrospinning, electrospray, and extrusion-based printing have been explored as manufacturing approaches to create nano-to-micrometric structures able to successfully encapsulate several bacteria types (e.g., *E. coli*, *Lactobacilli*). Although promising, the development of such bio-structures has been explored mainly from a microbiological standpoint by analyzing bacterial cell viability across time and different encapsulation parameters, such as time and mechano-electric forces. In fact, although electrospinning, electrospraying, and 3D printing have been demonstrated to be applicable processing technologies for probiotics, the full preservation of bacterial viability during the biofabrication processes and the subsequent storage remain the key aspects to be improved. As a future avenue, four-dimensional (4D) bioprinting could be increasingly exploited to address bacteriotherapy under a precise stimulating condition able to activate the printed biomaterial structure, such as, but not limited to, local change of pH [14], temperature, light, humidity, magnetic, mechanical and electrical forces [103,104]. Such stimuli can, in fact, promote a timely release of pro- and post-biotics to the targeted site, thus further improving the performance efficacy (Figure 10).

Finally, independently of the manufacturing approach, the current research has not fully characterized the biomaterial structures loaded with probiotics, thus necessitating additional investigation on the biomacromolecules used to create these structures, as well as the relationships between the physico-chemical, morphological, rheological, and mechanical properties. Such research fields could be considered as an intriguing path to improve the fabrication of specific devices for bacteriotherapy, which are designed to entrap, protect, and release bacterial cells or bacterial products according to the specific disease and body location. Furthermore, from a clinical perspective, additional experiments, both in vitro and in vivo, would be required to assess the effectiveness of the therapeutic approach, to better address the challenges of providing a targeted therapy, and to make bacteriotherapy a valuable antimicrobial treatment in real life. In vitro tests may avail themselves of advanced 3D in vitro healthy and pathologic tissue models, such as skin [105], intestine [106], lung [106], and tumor [107] models. 

## Figures and Tables

**Table 1:** Summary table on electrospinning probiotic-loaded polymers (SA: sodium alginate; PVA: polyvinyl alcohol; GI: gastrointestinal).

Probiotic	Polymer	Mechanical Properties	Targeted Application	Morphological Parameters	Ref.
*Lactobacillus acidophilus*	Arabic gum/PVA	Tensile strength = 14.21 ± 0.7 MPa Elongation at break = 27.8 ± 0.3%	GI	Diameter > 617 nm Thickness = 0.12 ± 0.01 mm	[45]
*Lactobacillus reuteri*	PVA	-	Functional fish fillets	Diameter = 381.83 ± 130.69 nm	[46]
*Lactobacillus plantarum*	SA/PVA	-	Food industry	Diameter = 270 ± 64 nm	[47]
*Lactobacillus paracasei*	SA/PVA	-	GI	Diameter = 842 ± 72 nm	[48]
*Lactobacillus rhamnosus*	PVA/PEC	-	Probiotic preservation	Diameter = from 112.30 ± 78.10 nm to 149.89 ± 25.66 nm	[30]

**Table 2:** Summary table on electrospraying of probiotic-loaded polymers (EA: egg albumen; WPI: whey protein isolate; PEO: polyethylene oxide; GI: gastrointestinal; URT: upper respiratory tract).

Probiotic	Polymer	Average Diameter	Targeted Application	Ref.
*Lactobacillus acidophilus*	Alginate/glycerol/zein	<550 μm	GI	[58]
*Lactobacillus acidophilus*	EA/SA	<700 μm	GI	[59]
*Lactobacillus plantarum*	Pectin	-	GI	[60]
*Lactobacillus plantarum*	Ca-alginate/chitosan	<500 μm	GI	[61]
*Lactobacillus plantarum, Bifidobacterium lactis*	Ca-alginate/chitosan	=710 μm	GI	[62]
*Lactobacillus acidophilus* LA-5	WPI/lactose	259–658 nm	-	[29]
*Lactobacillus plantarum* and others (commercial probiotic mixture)	SA/PEO	395 ± 23 μm	URT	[63]

**Table 3:** Summary table on 3D printing probiotic-loaded constructs (SA: sodium alginate; HA: hyaluronic acid; κ-CA: κ-carrageenan; GRAS: recognized as safe; FS: fumed silica; PAPN: Poly(ethylene glycol)-diacrylate/SA/poly(vinyl alcohol)/nanoclay; GI: gastrointestinal).

Probiotic/Bacterium	Polymer	Targeted Application	Mechanical Properties	Ref.
*Lactobacillus rhamnosus*	SA/gelatin	GI	Young’s moduli = 3.3 kPa (day 0) and 1.1 kPa (day 7)	[5]
*Escherichia coli*/*Bacillus subtilis*	Alginate	Infection prevention device	-	[79]
*Escherichia coli*	SA	Artificial biofilm	-	[80]
*Escherichia coli*	Alginate	Water filtration, metal ion sequestration, or civil engineering	-	[81]
*Shewanella oneidensis MR-1*	Alginate/cellulose	Microbial devices	-	[83]
*P. putida, X. xylinum*	HA/κ-CA/FS (Flink)	Biomedical/technological applications; biologically generated functional materials	G″ > G′ at strains > 10%; Viscosity < 10^8^ mPa·s Yield stress < 350 Pa	[84]
*Lactobacillus rhamnosus GG/Escherichia coli*	Eudragit^®^ L100 and S100	GI	-	[14]
*Lactobacillus rhamnosus CNCM I-4036*	GRAS proteins	GI	-	[85]
*Bifidobacterium animalis subsp. Lactis*	Mashed potatoes	GI	Yield stress 572–2558 Pa	[87]
*Lactobacillus crispatus*	Gelatin/alginate	Female genital tract	Viscosity = 1616 ± 19 mPa·s at 37 °C	[28]

## Data Availability

Data will be made available by the corresponding author upon request.
